# Effects of a 12-week dance intervention on left-behind children with co-occurring social anxiety and low self-concept

**DOI:** 10.3389/fpsyg.2025.1491743

**Published:** 2025-04-25

**Authors:** Xiaolin Li, Qian Yang, Zhenqian Zhou, Ming Zeng, Chunxia Lu, Weixin Dong

**Affiliations:** ^1^Department of Human Movement Science, Hunan Normal University, Changsha, Hunan, China; ^2^Department of Physical Education, Hunan Agricultural University, Changsha, Hunan, China; ^3^Department of Sport Psychology, Hunan International University, Changsha, Hunan, China

**Keywords:** left-behind children (LBC), dance intervention, social anxiety, self-concept, randomized controlled trial

## Abstract

**Objective:**

This study aimed to investigate the effects of a 12-week dance intervention on left-behind children (LBC) suffering from social anxiety and low self-concept, and to explore the relationship between social anxiety and self-concept.

**Methods:**

Sixty LBC who met the criteria were selected from a school in Shaoyang City, Hunan Province, and were randomly divided into an Interventional group (*n* = 30) and a Control group (*n* = 30). The Interventional group received a 45-min dance intervention five times a week for 12 weeks, while the Control group maintained their original lifestyle. Social anxiety and self-concept were measured three times using the Social Anxiety Scale for Children and the Piers-Harris Child Self-concept Scale: at baseline (T0), post-intervention (12 weeks, T1), and follow-up (14 weeks after baseline, T2).

**Results:**

(i) After the dance intervention, social anxiety and self-concept were significantly improved (*p* < 0.05). (ii) There was a significant negative correlation between the change scores (T1 minus T0) of social anxiety and self-concept (*p* < 0.05).

**Conclusion:**

Dance intervention is an acceptable, practical and effective intervention that we can incorporate into a health programme to improve social anxiety and low self-concept in LBC.

## 1 Introduction

As China's urbanization accelerates and economic transformation continues, a large number of rural laborers are migrating to cities seeking better employment opportunities and higher incomes. However, due to the limitations of the household registration system and disposable incomes, these laborers cannot bring their children to reside in urban arears (Zhang and Zheng, 2022). These minors, under the age of 16, who are left at home by their parents and cared for by other family members, are commonly referred to as left-behind children (LBC). According to the latest data, as of August 2023, the number of LBC at the compulsory education stage in China is 15.5 million, accounting for about 15% of all children in the country (Ministry of Education of the People's Republic of china, 2023). Previous research on LBC in China has found that although they benefit financially from the remittances sent home by their parents, they need to readjust to changes in the family dependency structure due to the lack of parental companionship over the years (Fellmeth et al., 2018). Over time, this prolonged separation fails to meet their emotional and psychological needs, making them more likely to develop various degrees of mental health problems compared to non-LBC (Tan et al., 2018; To et al., 2019). Among them, high social anxiety and low self-concept are the most prominent mental health problems observed in LBC (To et al., 2019).

It is noteworthy that social anxiety is the third most common mental health disorder after depression and substance abuse, with lifetime prevalence rates between 4 and 12% (Asnaani et al., 2010; Leigh and Clark, 2018; Stein, 2015). Age-of-onset data indicate that social anxiety typically occurs in late childhood and early adolescence, a period when individuals transition from familial dependence to peer interaction, shaping lifelong social skills (Bas-Hoogendam et al., 2018; Kilford et al., 2016). The development of specific neurocognitive abilities underpins this social reorientation (Kilford et al., 2016). One such ability is self-concept. As an important indicator of mental health, self-concept is significantly influenced by family environmental factors in its formation and development (Busch et al., 2021; Davis and Franzoi, 1986). Researchers have confirmed that specific groups of adolescents may be more susceptible to lower levels of self-concept than the general population (Ke et al., 2020). Additionally, some researchers have proposed a multidimensional model of self-concept from a developmental perspective, arguing that childhood is a critical period for its development (Larson and Richards, 1991).

Furthermore, research has found a link between social anxiety and self-concept (Kley et al., 2012; Nikolić et al., 2020). A pilot study of children with epilepsy by Jones et al. (2014) showed that when social anxiety symptoms were reduced in children with epilepsy, their levels of self-concept increased. Busch et al. (2021) demonstrated that self-concept was a significant predictor of anxiety symptom levels, with stronger inversed associations in adolescents. These findings provide evidential support for the relationship between social anxiety and self-concept in children, prompting us to explore whether the same association exists in LBC.

Currently, various non-pharmacological interventions targeting the underlying mechanisms to improve social anxiety and self-concept are showing positive results (Bennett et al., 2021; García-Martínez et al., 2012; Jazaieri et al., 2012). Among these, exercise intervention is one of the most commonly used and effective practical options, as it affects various physiological pathways related to social anxiety and self-concept, including improving angiogenesis and the balance of neurotransmitter concentrations, thus strengthening temporal cortices (LeBouthillier and Asmundson, 2017; Wassenaar et al., 2021). However, most studies on the beneficial effects of exercise interventions require specific equipment, venues, and aerobic or resistance training programs (LeBouthillier and Asmundson, 2017). These limitations hinder the accessibility and sustainability of exercise interventions. Additionally, these interventions are usually performed in isolation and lack interpersonal interaction, affecting participants' adherence to the intervention and psychosocial outcomes (He et al., 2022).

Given the potential benefits of exercise, a unique form of physical activity, dance intervention, was chosen to alleviate social anxiety and enhance self-concept in LBC. Dance was selected because it combines physical movement with social and musical elements (Yan et al., 2024). Unlike traditional exercise interventions, dance interventions include rhythmic movement coordination, balance, memory, social interaction, sound stimulation, and musical experience, which can improve compliance and psychosocial outcomes (Zhou et al., 2021). Moreover, the benefits of dance interventions being group activities, less site-demanding, easy to apply in various settings, and relatively inexpensive make them a potentially powerful intervention method (Huang et al., 2023; Liu et al., 2023). Previous studies have demonstrated that dance interventions improve physical and mental health indicators to a greater extent than other types of sport (Fong Yan et al., 2018).

However, although studies have explored the effects of dance interventions on the mental health of different populations, there are still limited intervention studies for LBC, and most of them focus on short-term effects. Therefore, this study is the first to use dance intervention, a novel approach, to track and analyze its effects on LBC's social anxiety and self-concept over a long period of time, and proposes the following hypotheses: (i) Dance intervention can effectively alleviate social anxiety in LBC; (ii) Dance intervention can effectively enhance self-concept in LBC; (iii) There is a correlation between the amount of change in social anxiety and self-concept.

## 2 Methods

### 2.1 Study design and procedure

**Figure 1:**
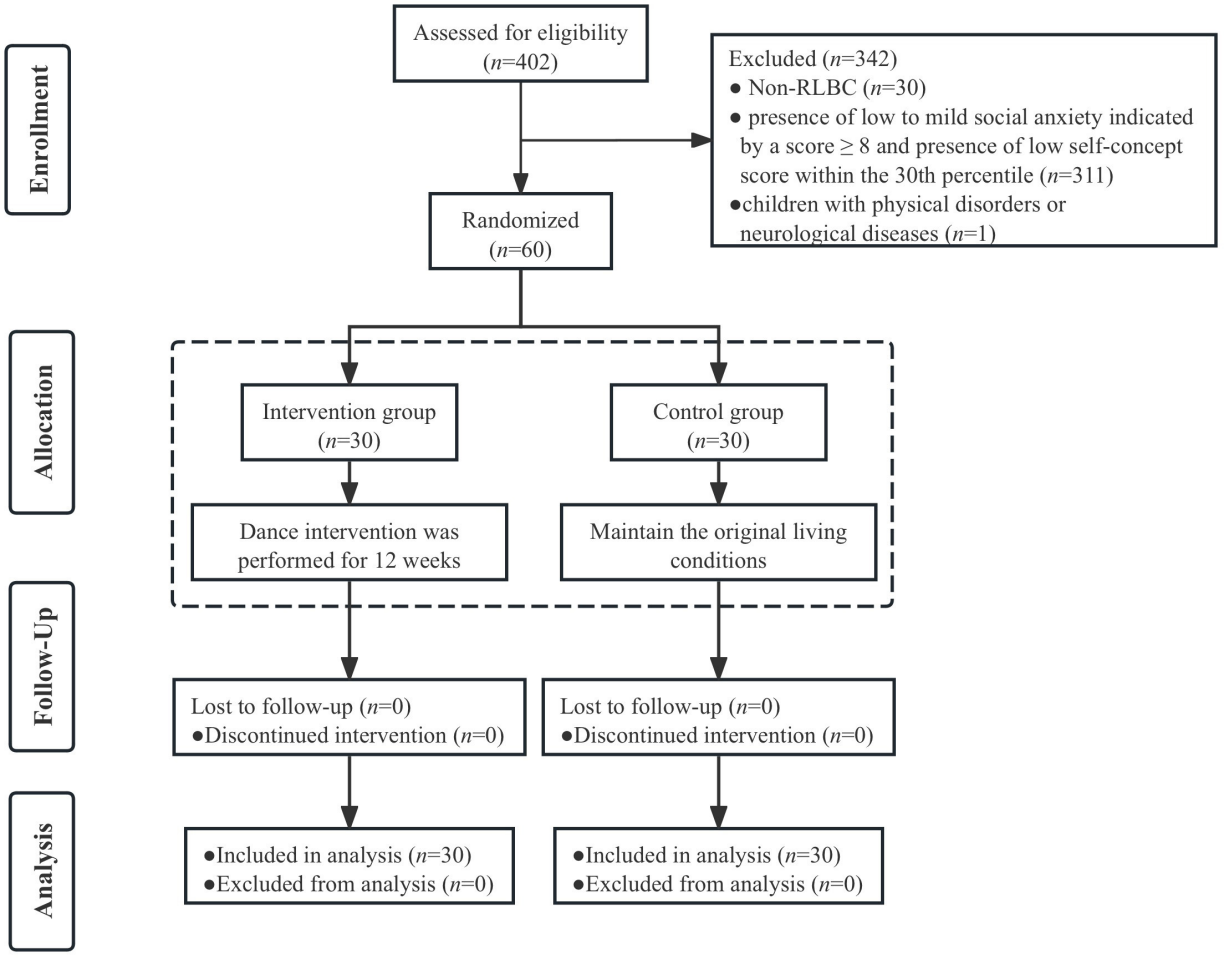
CONSORT flow diagram.

The study was a randomized controlled trial (RCT) and was assessed at three time points: baseline (T0), post-intervention (12 weeks, T1), and follow-up (14 weeks after baseline, T2). The trial protocol adhered to the principles of the Declaration of Helsinki and was approved by the Medical Research Ethics Committee of Hunan Normal University (Registered under No. 2022039). Parents or legal guardians of all participants provided informed consent after the details of the study were fully explained. The CONSORT flow diagram of this study is shown in Figure 1.

### 2.2 Participants

Participants were recruited from a school specializing in the education of LBC in Shaoyang City, Hunan Province. A total of 402 students (from year three to year seven) were initially considered. The eligibility criteria were as follows: (i) inclusion criteria: presence of low to mild social anxiety indicated by a score ≥ 8; presence of low self-concept score within the 30th percentile (equivalent to a score of 46). (ii) exclusion criteria: children from single-parent families or orphans; children with physical disorders or neurological diseases. Ultimately, 60 eligible LBC were randomly assigned to either the Intervention group (*n* = 30) or the Control group (*n* = 30).

### 2.3 Dance intervention

The intervention group participated in dance training four times a week, each session lasting 45 minutes (from 14:10 to 14:55 p.m., Monday to Friday). Each training session consisted of three parts: warm-up (5 min), dance training (35 min), and relaxation (5 min). The warm-up activities included exercises for the head, neck, shoulder, waist, hip, leg, wrist, and ankle. The dance training was based on the Cha Cha Cha style of Latin dance and was divided into three phases. The first phase focused on basic Cha Cha Cha movements, the second phase involved solo Cha Cha Cha combinations, and the third phase concentrated on partner Cha Cha Cha combinations. The relaxation exercises included stretches for the shoulders, neck, arm, back, and legs, as well as breathing and relaxation techniques. Details of the intervention programme can be found in Supplementary material. Two dance teachers with more than 5 years of experience in dance intervention conducted the dance sessions. The intensity of the exercise was maintained between 60 and 80% of the participants' maximum heart rate. A heart rate monitor (Polar H9; Polar Electro Oy Inc; Fi) was used to monitor the participants' heart rate intensity. The control group maintained their original lifestyle throughout the study period.

### 2.4 Measures

#### 2.4.1 Basic information questionnaire

At baseline, the children completed a questionnaire that gathered information about their age, grade, gender, parent-children separation form, parent-children separation duration, guardianship type, and frequency of contact with the absent parent. This information helped us to develop and implement the intervention program. For example, the Cha Cha Cha dance was selected as the most appropriate dance for the age of the participants, and partner dances and group dances requiring duets were implemented in the third phase of the intervention according to the gender of the participants (He et al., 2022).

#### 2.4.2 Social anxiety scale for children (SASC)

The Social Anxiety Scale for Children (SASC) was used to assess the social anxiety of LBC (La Greca, 1988). This scale is suitable for children and adolescents aged 7–16 years. It contains 10 items, including two factors: Fear of Negative Evaluation (FNE), and Social Avoidance and Distress (SAD). Each item uses a 3-point scoring system: 0 for never, 1 for sometimes, and 2 for often, with a total score of 20. According to the original scale, a score ≥ 8 indicates social anxiety, with higher scores representing more severe anxiety. The SASC has been translated into Chinese, and its psychometric properties have been validated in a large Chinese sample, demonstrating high reliability and validity (Mo and Bai, 2023; Wang et al., 2011). Therefore, this study used the Chinese version of the SASC to assess social anxiety in Chinese children. The Cronbach's α coefficient of the scale was 0.721, and the KMO value was 0.813.

#### 2.4.3 Piers-harris child self-concept scale (PHCSS)

The Piers-Harris Child Self-concept Scale (PHCSS) was used to assess the self-concept of LBC (Pier, 1964). This 80-item self-report measure evaluates self-concept in children and adolescents aged 7–18 years. Each question has a standard “yes” or “no” answer. The scale is divided into six factors: Behavior Adjustment (BEH), Intellectual and School Status (INT), Physical Appearance and Attributes (PHY), Freedom and Anxiety (FRE), Popularity (POP), and Happiness and Satisfaction (HAP). According to the original scale, scores below the 30th percentile indicate a low level of self-concept. The Chinese version of the PHCSS is reported to be a reliable and valid measure among Chinese children (Huang et al., 2021). Therefore, this study used the Chinese version of the PHCSS to assess the self-concept of Chinese children. The Cronbach's α coefficient of the scale was 0.889 and the KMO value was 0.825.

### 2.5 Statistical analyzes

All statistical analyzes were conducted using SPSS Statistics software (release 29.0; IBM Corp., Armonk, NY, USA). The normal distribution of the outcome measures was confirmed using the Shapiro–Wilk normality test. Chi-square and independent *t*-test were used to determine baseline differences between the individuals randomly assigned to the Intervention group and the Control group. A repeated measures analysis of covariance (ANCOVA) was performed to examine the intervention effects of dance therapy on social anxiety and self-concept in LBC, with age included as a covariate in the analytical model. Pearson correlations were used to analyze the association between social anxiety and self-concept change scores (T1 minus T0). *P*-values less than 0.05 were considered statistically significant.

## 3 Results

### 3.1 Baseline characteristics

Out of the 402 LBC surveyed, 60 were identified as suffering from both social anxiety and low self-concept, accounting for 15 percent of all children. At baseline, no significant differences were observed in sociodemographic variables, social anxiety, and self-concept among these 60 LBC (*p* > 0.05). Participants' characteristics are presented in Table 1.

**Table 1:** Basic characteristics of participants at baseline.

**Variables**	**Category**	**Intervention group (*n* = 30) [*n* (%)/M ±SD]**	**Control group (*n* = 30) [*n* (%)/M ±SD]**	***x*^2^/*t***	***P***
Gender	Male	15 (50.0%)	18 (60.0%)	0.606	0.436
	Female	15 (50.0%)	12 (40.0%)		
Grade	3	7 (23.3%)	3 (10.0%)	2.532	0.639
	4	2 (6.7%)	1 (3.3%)		
	5	4 (13.3%)	5 (16.7%)		
	6	5 (16.7%)	7 (23.3%)		
	7	12 (40.0%)	14 (46.7%)		
Parent-children separation form	Father	4 (13.3%)	8 (26.7%)	2.933	0.231
	Mother	7 (23.3%)	3 (10.0%)		
	Parents	19 (63.3%)	19 (63.3%)		
Parent-children separation duration (month)	6–12	14 (46.7%)	14 (46.7%)	1.714	0.634
	13–24	5 (16.7%)	2 (6.7%)		
	25–36	9 (30.0%)	12 (40.0%)		
	> 36	2 (6.7%)	2 (6.7%)		
Guardianship type	Father	3 (10.0%)	1 (3.3%)	2.239	0.524
	Mother	6 (20.0%)	5 (16.7%)		
	Grandfather/ Grandmother	11 (36.7%)	16 (53.3%)		
	Others	10 (33.3%)	8 (26.7%)		
Frequency of contact with the absent parent (time/month)	< 1	17 (56.7%)	14 (46.7%)	1.322	0.724
	1–3	7 (23.3%)	11 (36.7%)		
	4–14	4 (13.3%)	3 (10.0%)		
	≥ 15	2 (6.7%)	2 (6.7%)		
Age		10.73 ± 1.99	11.20 ± 1.24	−1.086	0.283
SASC		11.30 ± 3.25	10.30 ± 2.54	1.329	0.189
FNE		6.70 ± 2.38	6.13 ± 2.06	0.985	0.329
SAD		4.60 ± 1.45	4.17 ± 1.42	1.170	0.247
PHCSS		37.70 ± 7.26	38.00 ± 7.00	−0.163	0.871
BEH		10.03 ± 2.51	9.30 ± 2.98	1.030	0.307
INT		6.23 ± 3.12	6.40 ± 2.66	−0.223	0.824
PHY		3.33 ± 2.29	4.33 ± 2.22	−1.716	0.091
FRE		5.73 ± 2.79	6.10 ± 2.52	−0.534	0.596
POP		6.60 ± 2.55	7.10 ± 1.92	−0.857	0.395
HAP		5.03 ± 1.90	5.47 ± 1.74	−0.921	0.361

M, Mean; SD, Standard Deviation; SASC, Social Anxiety Scale for Children; FNE, Fear of Negative Evaluation; SAD, Social Avoidance and Distress; PHCSS, Piers-Harris Child Self-concept Scale; BEH, Behavior Adjustment; INT, Intellectual and School Status; PHY, Physical Appearance and Attributes; FRE, Freedom and Anxiety; POP, Popularity; HAP, Happiness and Satisfaction.

### 3.2 Effects of intervention

#### 3.2.1 Changes of social anxiety in LBC

The results of the repeated measures ANOVA indicated a significant interaction effect for the SASC [*F*_(2, 56)_ = 17.131, $\eta_{p}^{2}$ = 0.380, *p* < 0.001], FNE [*F*_(2, 56)_ = 7.851, $\eta_{p}^{2}$= 0.219, *p* < 0.001], and SAD [*F*_(2, 56)_ = 7.851, $\eta_{p}^{2}$= 0.219, *p* < 0.001]. Therefore, further simple effects analyzes were conducted.

The results showed a significant difference in SASC, FNE, and SAD (all *p* < 0.001) between the intervention and control groups at T1; At T2, there was a significant difference in SASC (*p* = 0.023), FNE (*p* = 0.032), but no significant difference in SAD (*p* = 0.144) between the intervention and control groups.

**Figure 2:**
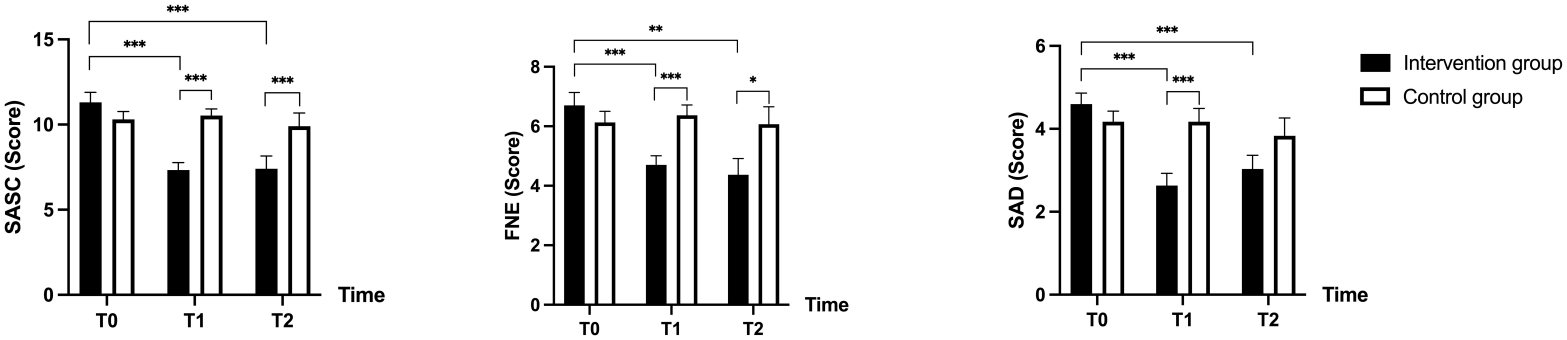
Trends in social anxiety in both groups before and after the intervention. SASC, Social Anxiety Scale for Children; FNE, Fear of Negative Evaluation; SAD, Social Avoidance and Distress. T0, at baseline; T1, post-intervention (12 weeks); T2, follow-up (14 weeks after baseline). *, p < 0.05; **, p < 0.01; ***, p < 0.001.

Within the intervention group, significant differences were observed from T0 to T1 for SASC, FNE, and SAD (all *p* < 0.001). However, no significant differences were found from T1 to T2 for SASC (*p* = 0.925), FNE (*p* = 0.878), and SAD (*p* = 0.812). Significant differences were again noted from T0 to T2 for SASC (*p* < 0.001), FNE (*p* = 0.002), and SAD (*p* < 0.001). Conversely, in the control group, there were no significant differences between any of the three time points for SASC, FNE, and SAD (all *p* = 1.00). Table 2 and Figure 2 presents a trend plot of the total social anxiety score and the factor scores.

**Table 2:** Effects of dance intervention on social anxiety.

**Variables**	**Groups**	**T0** **(M ±SD)**	**T1 (M ±SD)**	**T2** **(M ±SD)**	**Time**	**Group**	Time^*^Group
SASC	Intervention group	11.30 ± 3.25	7.33 ± 2.41	7.40 ± 2.13	*F* = 0.452 *P* = 0.638 *$\eta_{p}^{2}$*= 0.076	*F* = 5.583 *P* = 0.022 *$\eta_{p}^{2}$*= 0.089	*F* = 17.131 *P* < 0.001 *$\eta_{p}^{2}$*= 0.380
	Control group	10.30 ± 2.54	10.53 ± 2.10	9.90 ± 2.27			
FNE	Intervention group	6.70 ± 2.38	4.70 ± 1.71	4.37 ± 1.99	*F* = 0.183 *P* = 0.833 *$\eta_{p}^{2}$*= 0.067	*F* = 3.535 *P* = 0.065 *$\eta_{p}^{2}$*= 0.068	*F* = 7.851 *P* < 0.001 *$\eta_{p}^{2}$*= 0.219
	Control group	6.13 ± 2.06	6.37 ± 1.90	6.07 ± 1.21			
SAD	Intervention group	4.60 ± 1.45	2.63 ± 1.63	3.30 ± 1.81	*F* = 0.183 *P* = 0.833 *$\eta_{p}^{2}$*= 0.087	*F* = 3.535 *P* = 0.065 *$\eta_{p}^{2}$*= 0.068	*F* = 7.851 *P* < 0.001 *$\eta_{p}^{2}$*= 0.219
	Control group	4.17 ± 1.42	4.17 ± 1.78	3.83 ± 1.38			

M, mean; SD, standard deviation; SASC, social anxiety scale for children; FNE, fear of negative evaluation; SAD, social avoidance and distress.

#### 3.2.2 Changes of self-concept in LBC

The results of repeated measures ANOVA indicated a significant interaction effect for POP [*F*_(2, 56)_ = 3.627, $\eta_{p}^{2}$ = 0.060, *p* = 0.032] and HAP [*F*_(2, 56)_ = 4.750, $\eta_{p}^{2}$ = 0.077, *p* = 0.011]. Therefore, further simple effects analyzes were conducted.

**Figure 3:**
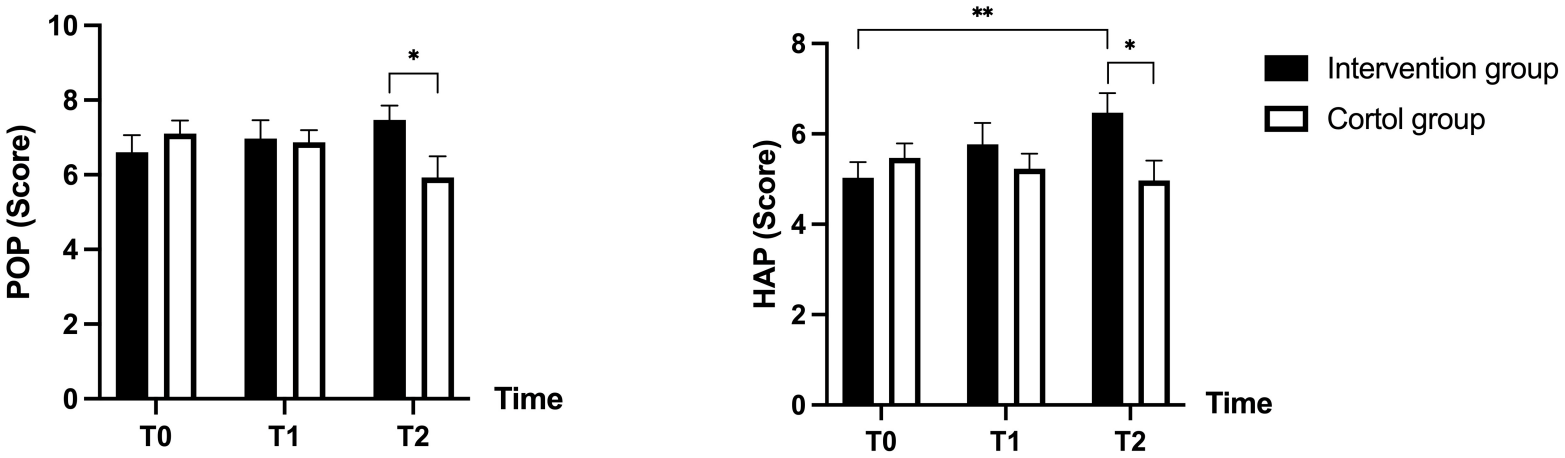
Trends in self-concept scores in both groups before and after the intervention. POP, Popularity; HAP, Happiness and Satisfaction. T0, at baseline; T1, post-intervention (12 weeks); T2, follow-up (14 weeks after baseline). *, *p* < 0.05; **, *p* < 0.01.

The results showed a significant difference in POP (*p* = 0.021) and HAP (*p* = 0.014) between the intervention and control groups at T2. Additionally, within the intervention group, HAP showed significant differences from T0 to T2 (*p* = 0.006). Table 3 and Figure 3 presents a trend plot of the total self-concept score and the factor scores.

**Table 3:** Effects of dance intervention on self-concept.

**Variables**	**Groups**	**T0** **(M ±SD)**	**T1 (M ±SD)**	**T2** **(M ±SD)**	**Time**	**Group**	Time^*^Group
PHCSS	Intervention group	37.70 ± 7.26	40.87 ± 11.09	39.50 ± 10.87	*F* = 0.710 *P* = 0.496 *$\eta_{p}^{2}$*= 0.025	*F* = 0.131 *P* = 0.718 *$\eta_{p}^{2}$*= 0.071	*F* = 0.884 *P* = 0.419 *$\eta_{p}^{2}$*= 0.061
	Control group	38.00 ± 7.00	38.27 ± 7.48	37.57 ± 14.76			
BEH	Intervention group	10.03 ± 2.51	10.27 ± 3.74	11.17 ± 3.42	*F* = 0.451 *P* = 0.623 *$\eta_{p}^{2}$*= 0.058	*F* = 3.303 *P* = 0.074 *$\eta_{p}^{2}$*= 0.055	*F* = 1.567 *P* = 0.213 *$\eta_{p}^{2}$*= 0.077
	Control group	9.30 ± 2.98	9.50 ± 2.54	8.93 ± 4.14			
INT	Intervention group	6.23 ± 3.12	7.40 ± 3.69	7.60 ± 3.03	*F* = 0.283 *P* = 0.749 *$\eta_{p}^{2}$*= 0.035	*F* = 0.485 *P* = 0.489 *$\eta_{p}^{2}$*= 0.068	*F* = 1.443 *P* = 0.241 *$\eta_{p}^{2}$*= 0.025
	Control group	6.40 ± 2.66	6.33 ± 3.20	6.60 ± 3.76			
PHY	Intervention group	3.33 ± 2.29	5.10 ± 3.56	4.57 ± 3.06	*F* = 1.043 *P* = 0.354 *$\eta_{p}^{2}$*= 0.018	*F* = 0.679 *P* = 0.413 *$\eta_{p}^{2}$*= 0.012	*F* = 2.936 *P* = 0.059 *$\eta_{p}^{2}$*= 0.049
	Control group	4.33 ± 2.22	4.30 ± 2.25	5.10 ± 3.17			
FRE	Intervention group	5.73 ± 2.79	5.83 ± 3.45	5.73 ± 3.29	*F* = 1.361 *P* = 0.265 *$\eta_{p}^{2}$*= 0.046	*F* = 0.329 *P* = 0.569 *$\eta_{p}^{2}$*= 0.046	*F* = 0.444 *P* = 0.644 *$\eta_{p}^{2}$*= 0.016
	Control group	6.10 ± 2.52	6.67 ± 2.82	5.40 ± 3.19			
POP	Intervention group	6.60 ± 2.55	6.97 ± 2.68	7.47 ± 2.11	*F* = 0.294 *P* = 0.733 *$\eta_{p}^{2}$*= 0.045	*F* = 0.497 *P* = 0.484 *$\eta_{p}^{2}$*= 0.029	*F* = 3.627 *P* = 0.032 *$\eta_{p}^{2}$*= 0.660
	Control group	7.10 ± 1.92	6.87 ± 1.80	5.93 ± 3.11			
HAP	Intervention group	5.03 ± 1.90	5.77 ± 2.61	6.47 ± 2.39	*F* = 0.251 *P* = 0.776 *$\eta_{p}^{2}$*= 0.034	*F* = 1.032 *P* = 0.314 *$\eta_{p}^{2}$*= 0.018	*F* = 4.750 *P* = 0.011 *$\eta_{p}^{2}$*= 0.077
	Control group	5.47 ± 1.74	5.23 ± 1.83	4.97 ± 2.40			

M, mean; SD, standard deviation; PHCSS, piers-harris child self-concept scale; BEH, behavior adjustment; INT, intellectual and school status; PHY, physical appearance and attributes; FRE, freedom and anxiety; POP, popularity; HAP, happiness and satisfaction.

### 3.3 Correlations between social anxiety and self-concept change scores

Table 4 presents the change scores for each group, indicating significant reductions in social anxiety scores (SASC, FNE, SAD) in the intervention and combined groups, but not in the control group. Moreover, self-concept showed significant improvement in the intervention and combined groups (PHCSS, BEH, INT, PHY, FRE, POP, HAP), while no significant effects were detected for the four factors (INT, PHY, POP, HAP) in the control group.

**Table 4:** Change in social anxiety and self-concept scores before and after the intervention.

**Variables**	**Intervention group (*n* = 30) [M ±SD]**	**Control group (*n* = 30) [M ±SD]**	**Both groups (*n* = 60) [M ±SD]**
SASC	−3.97 ± 3.49	0.23 ± 1.74	−1.87 ± 3.44
FNE	−2.00 ± 2.64	0.23 ± 1.65	−0.88 ± 2.46
SAD	−1.97 ± 1.79	0.45 ± 1.26	−0.98 ± 1.83
PHCSS	3.17 ± 9.84	0.27 ± 5.61	1.72 ± 8.05
BEH	0.23 ± 3.27	0.20 ± 2.57	0.22 ± 2.91
INT	1.17 ± 3.24	−0.07 ± 3.34	0.55 ± 3.32
PHY	1.77 ± 2.94	−0.03 ± 2.46	0.87 ± 2.84
FRE	0.10 ± 3.13	0.57 ± 2.32	0.33 ± 2.74
POP	0.37 ± 3.33	−0.23 ± 1.92	0.07 ± 2.71
HAP	0.73 ± 2.60	−0.23 ± 1.78	0.25 ± 2.60

Note: SASC, Social Anxiety Scale for Children; FNE, Fear of Negative Evaluation; SAD, Social Avoidance and Distress; PHCSS, Piers-Harris Child Self-concept Scale; BEH, Behavior Adjustment; INT, Intellectual and School Status; PHY, Physical Appearance and Attributes; FRE, Freedom and Anxiety; POP, Popularity; HAP, Happiness and Satisfaction.

**Figure 4:**
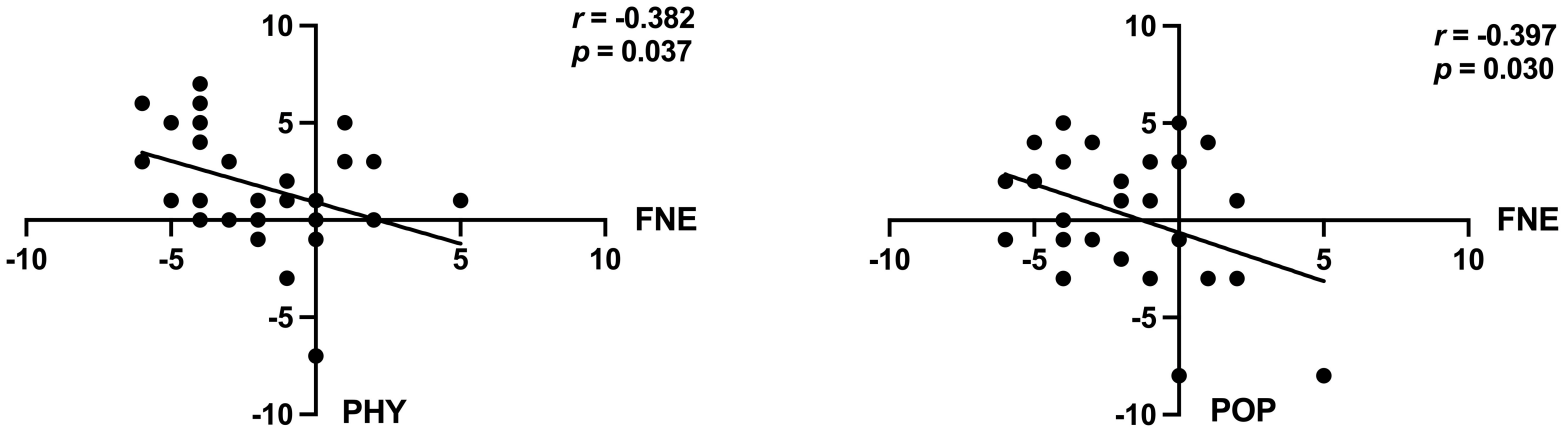
Trends in correlations between social anxiety and self-concept in the intervention group. FNE, Fear of Negative Evaluation; PHY, Physical Appearance and Attributes; POP, Popularity.

In order to verify the relationship between social anxiety and self-concept, the change scores of the post-test data (T0) minus the pre-test data (T1) were analyzed. The correlation results indicated a significant negative correlation between FNE and PHY (*p* = 0.037), POP (*p* = 0.030) in the intervention group as can be seen in Table 5 and Figure 4, however the control group did not show any significant correlations.

**Table 5:** Correlations between social anxiety and self-concept.

**Groups**	**Variables**	**PHCSS**	**BEH**	**INT**	**PHY**	**FRE**	**POP**	**HAP**
Intervention group (*n* = 30)	SASC	−0.068	0.255	−0.185	−0.304	−0.808	−0.223	−0.252
	FNE	−0.207	0.120	−0.222	−0.382^*^	−0.138	−0.397^*^	−0.211
	SAD	0.174	0.258	−0.031	−0.025	0.049	0.154	−0.176
Control group (*n* = 30)	SASC	0.263	0.222	−0.140	0.188	0.275	−0.035	0.209
	FNE	0.238	0.143	−0.047	0.146	0.099	0.007	0.160
	SAD	0.049	0.117	−0.131	0.067	0.248	−0.057	0.077
Both groups (*n* = 60)	SASC	−0.107	0.170	−0.239	−0.312^*^	0.065	−0.210	−0.235
	FNE	−0.161	0.110	−0.216	−0.314^*^	−0.016	−0.306^*^	−0.185
	SAD	0.015	0.171	−0.161	−0.166	0.144	0.017	−0.194

SASC, social anxiety scale for children; FNE, fear of negative evaluation; SAD, social avoidance and distress; PHCSS, Piers-Harris child self-concept scale; BEH, behavior adjustment; INT, intellectual and school status; PHY, physical appearance and attributes; FRE, freedom and anxiety; POP, popularity; HAP, happiness and satisfaction. ^*^p < 0.05.

**Figure 5:**
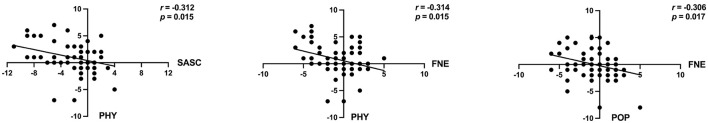
Trends in correlations between social anxiety and self-concept in both groups. Note: SASC, Social Anxiety Scale for Children; FNE, Fear of Negative Evaluation; PHY, Physical Appearance and Attributes; POP, Popularity.

Additionally, for both control and intervention groups combined, there was a significant negative correlation between SASC and PHY (*p* = 0.015), and a significant negative correlation between FNE and PHY (*p* = 0.015), and FNE and POP (*p* = 0.017). Table 5 and Figure 5 describe in detail the changes for the combined group.

## 4 Discussion

The study results indicate that the dance intervention effectively alleviated social anxiety and enhanced self-concept among LBC. Furthermore, a significant correlation was observed between improvements in social anxiety and self-concept from baseline to post- intervention, supporting our initial hypothesis.

After 12 weeks of dance intervention, total SASC scores (including FNE and SAD subscales) decreased significantly and remained stable at the 14-week follow-up. These findings align with prior research. For instance, Liu et al. (2023) demonstrated that Latin dance fosters social connections and reduces anxiety, while Bennett et al. (2021) concluded dance interventions effectively mitigate anxiety symptoms across populations. The efficacy of dance in alleviating social anxiety symptoms in LBC can be attributed to several factors. Dance is a social activity rooted in human culture, which helps individuals improve their social skills and reduce social isolation by engaging in group activities with shared interests and goals (Liu et al., 2021). In our intervention, the inclusion of paired dancing in Phase 3 increased interaction opportunities among LBC, promoting socialization and peer interaction, which likely alleviated social anxiety. Additionally, dance as an aerobic exercise promotes the release of endorphins and dopamine, contributing to reduced social anxiety (Feenstra et al., 2022). Dance also combines physical activity with music, providing a sense of pleasure and relaxation (Särkämö et al., 2013). Neuroimaging studies have shown that music activates various brain areas, including the amygdala and mesolimbic reward system, which can profoundly affect emotions like anxiety and depression (de Witte et al., 2022).

The 12-week intervention significantly increased PHCSS total scores, particularly in POP and HAP. Although there are limited studies on dance interventions for self-concept, some research has shown positive outcomes. For instance, Ren et al. (2021) found that dance improved self-concept and emotional expression in individuals with chronic diseases. A meta-analysis by Koch et al. (2019) also reported positive effects of dance on various self-concept indicators. The mechanisms by which dance improves self-concept include promoting neuroplasticity in brain regions responsible for motor control, emotional regulation, and cognitive functions through repeated practice and challenges (Shim et al., 2019; Van der Aar et al., 2022). Additionally, the deep breathing and rhythmic movements in dance activate the vagus nerve, promoting parasympathetic activity and physiological relaxation, aiding in self-reflection and introspection (Christensen et al., 2021). Dance also integrates multiple sensory inputs, enhancing the brain's ability to process information and improving self-concept (Kronsted, 2020). However, it is particularly worth highlighting that in the self-concept intervention, the POP and HOP factors showed delayed improvement only 2 weeks after the intervention. Given that improvement occurred after 2 weeks rather than immediately after the intervention, the time lag seems to imply that there may be mediators of this delayed effect (Ho et al., 2020). Social anxiety or other negative emotional and psychological factors may have temporally mediated the effects of the intervention on self-concept.

Our findings highlight a correlation between the amount of change in social anxiety and self-concept from T0 to T1. Previous studies have corroborated this relationship. Lindfors et al. (2014) found that self-concept influenced anxiety symptoms and work ability outcomes over 3 years of psychotherapy for anxiety disorders. Kley et al. (2012) identified self-concept as a predictor of outcome in social anxiety treatment for children and adolescents. The relationship between social anxiety and self-concept can be traced back to the cognitive model, which posits that socially anxious individuals are driven by the desire to make a good impression but doubt their ability to do so, leading to heightened self-focus and negative self-concept (Gilboa-Schechtman et al., 2017; Goldin et al., 2021). In addition, Spurr and Stopa (2002) view this relationship from an evolutionary perspective, emphasizing the importance of self-concept content based on social hierarchy and affiliation systems. Overall, various models emphasize the interdependence of social anxiety and self-concept (Hofmann, 2007).

There are some limitations to our study. First, the relatively short follow-up period and the small sample size, limited to LBC from the Hunan region, may restrict the generalizability of our findings to other regions in China. Second, our assessments relied on self-reported scales, which, despite their reliability, may be subject to bias and may not be as accurate as objective measures such as cortisol levels, heart rate variability, and other physiological indicators (Van der Aar et al., 2022). Third, in the absence of an otherwise active control group, the improvements in social anxiety and self-concept that we observed in the intervention group may have been related to an increase in weekly social interactions rather than a direct effect of the dance intervention itself. Therefore, further clarification of the specific effects of the dance intervention on these psychological variables is needed in subsequent studies, particularly to distinguish the relative roles of dance activities and social interactions in improving social anxiety and self-concept in the LBC.

## 5 Conclusions

This study provides empirical support for the use of dance interventions in the mental health of LBC, demonstrating that they are effective in improving social anxiety and self-concept. The study is also highly generalizable and can be widely applied in schools and other settings, which is highly relevant. Future research could further explore the effectiveness of combining different types of dance and other mental health interventions to enhance the psychological wellbeing of the LBC population.

## Data Availability

The original contributions presented in the study are included in the article/Supplementary material, further inquiries can be directed to the corresponding author.
