# Supplementary figures and images for: Effects of a 12-week dance intervention on left-behind children with co-occurring social anxiety and low self-concept
Source: Front Psychol. 2025 Apr 25;16:1491743. doi: 10.3389/fpsyg.2025.1491743 (PMC12062174; doi:10.3389/fpsyg.2025.1491743)

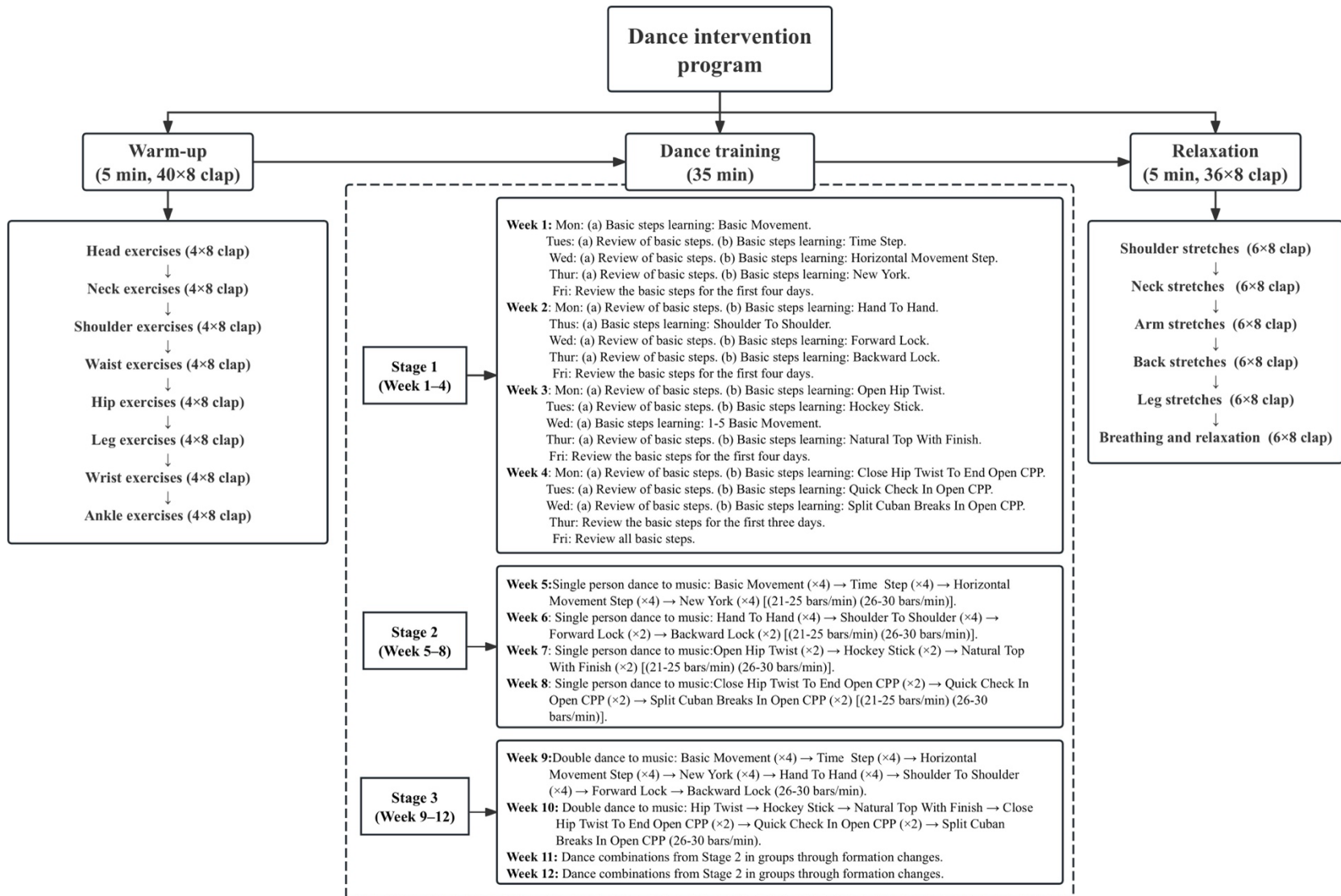

**Supplementary Figure 1.** Detailed intervention programme for the dance intervention group.

Supplement: Supplementary file 1 [file Data_Sheet_1.pdf]
